# Supplementary material for: Inhibition of Sphingosine Kinase 2 Results in PARK2-Mediated Mitophagy and Induces Apoptosis in Multiple Myeloma
Source: Curr Oncol. 2023 Mar 4;30(3):3047–63. doi: 10.3390/curroncol30030231 (PMC10047154; doi:10.3390/curroncol30030231)
Supplement: Supplementary file 1 [file curroncol-30-00231-s001.zip › curroncol-2250005-supplementary.pdf]

Current Oncology

MDPI

Supplementals

Inhibition of Sphingosine Kinase 2 Results in PARK2-Mediated Mitophagy and Induces Apoptosis in Multiple Myeloma

Jian Wu, Shengjun Fan, Daniel Feinberg, Xiaobei Wang, Shaima Jabbar and Yubin Kang *

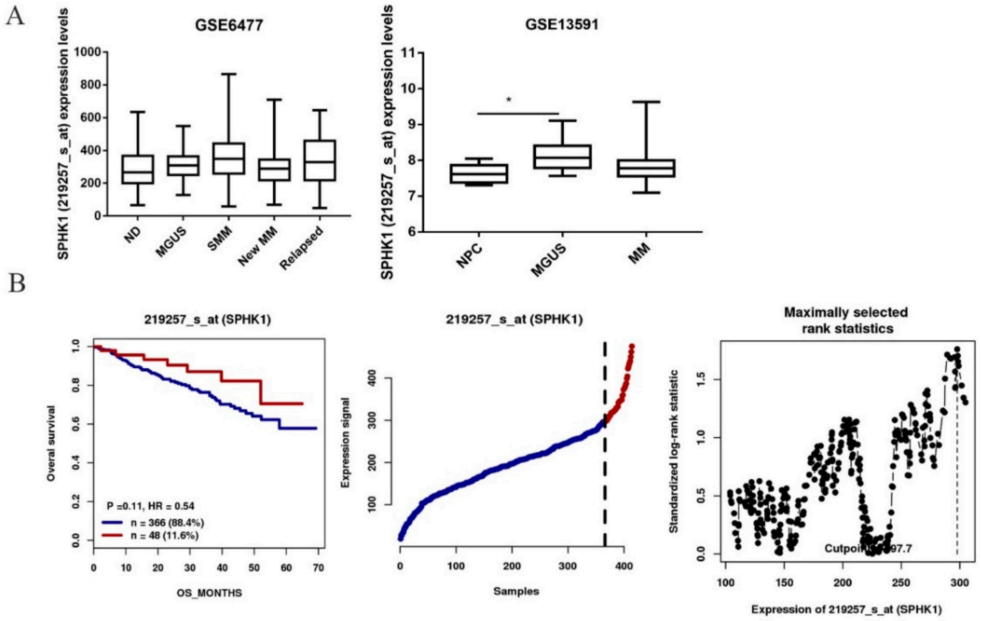

Figure S1. SK1 (219257_s_at) expression in different stages of MM and the relationship with overall survival.

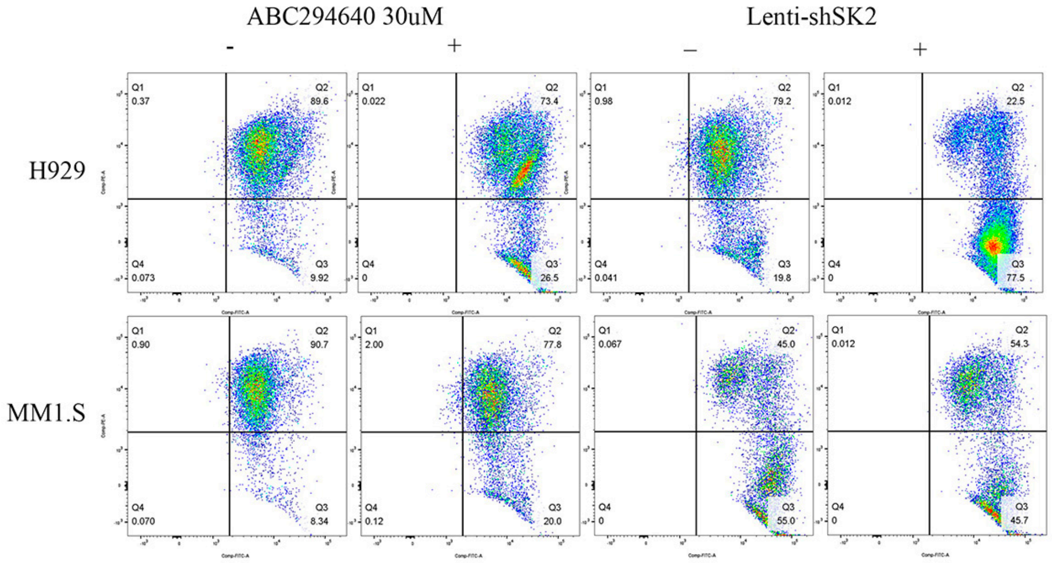

Figure S2. Inhibition SK2 expression through ABC294640 or Lenti-SK2 result to mitochondrial membrane potential changed. Flow cytometric analysis of H929 and MM1.S using the MitoProbe JC-1 assay kit.

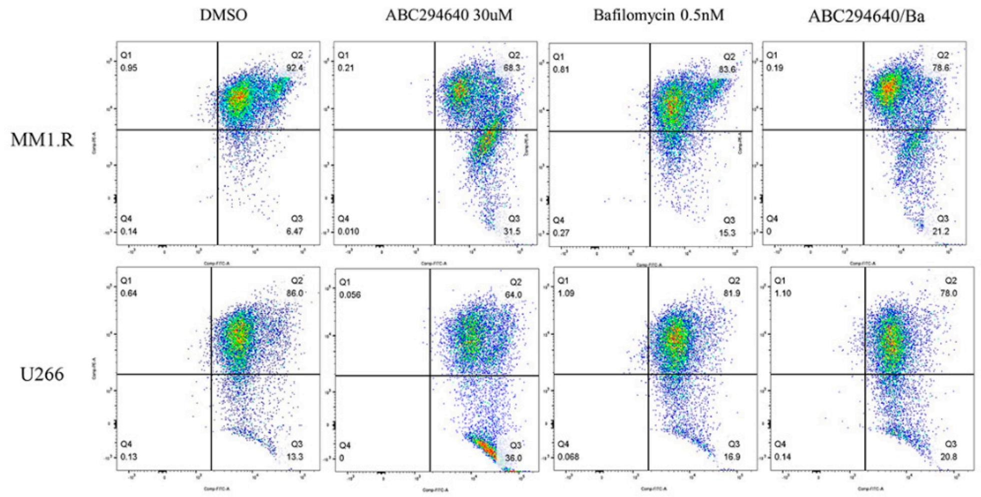

Figure S3. Using Bafilomycin reduced the ABC294640-induced mitophagy. Combination ABC294640 with bafilomycin in MM1.R and U266 48h, JC-1 assay was performed to display the mitochondrial membrane potential.

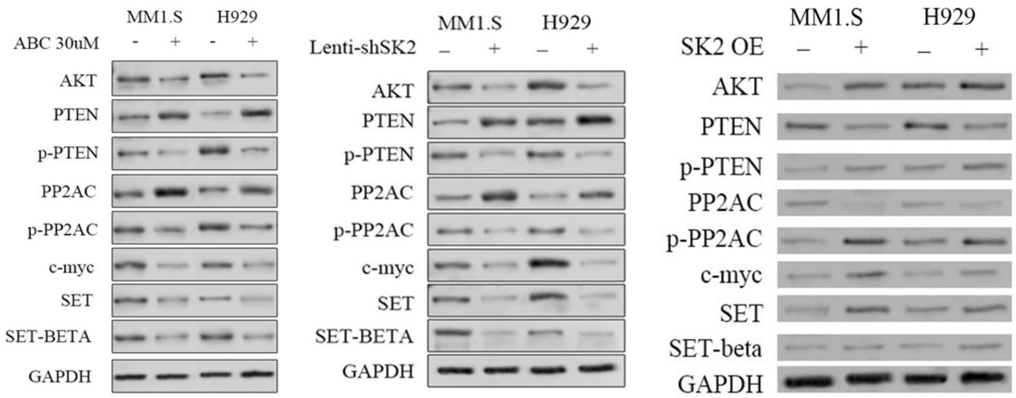

Figure S4. Inhibition SK2 involved AKT/c-Myc/SET pathway. ABC294640 or Lenti-shsk2 were used to inhibit SK2 expression in MM1S and H929 cell lines. Protein lysates were subjected to western blot with indicated antibodies (left and middle). MM1.S and H929 were transfected with SK2 plasmid 48h, total protein lysates were subjected to western blot with indicated antibodies(right).

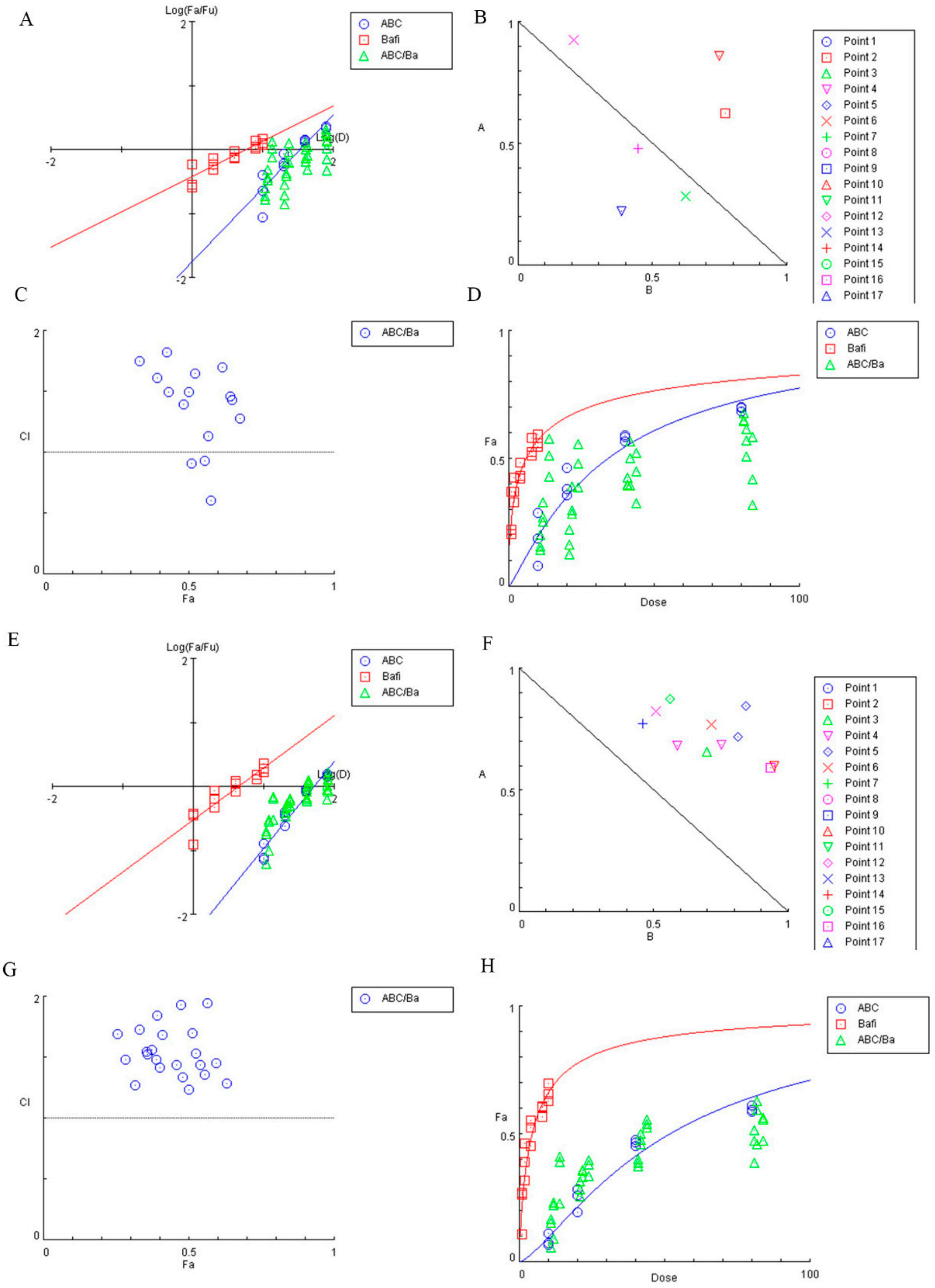

Figure S5. The graphic representations obtained from the CompuSyn Report for ABC294640 and Bafilomycin in MM1.R and U266. (A, E) Median-effect plot; (B, F) Isobologrms; (C, G) Combination index plot; (D, H) Dose-effect curve.

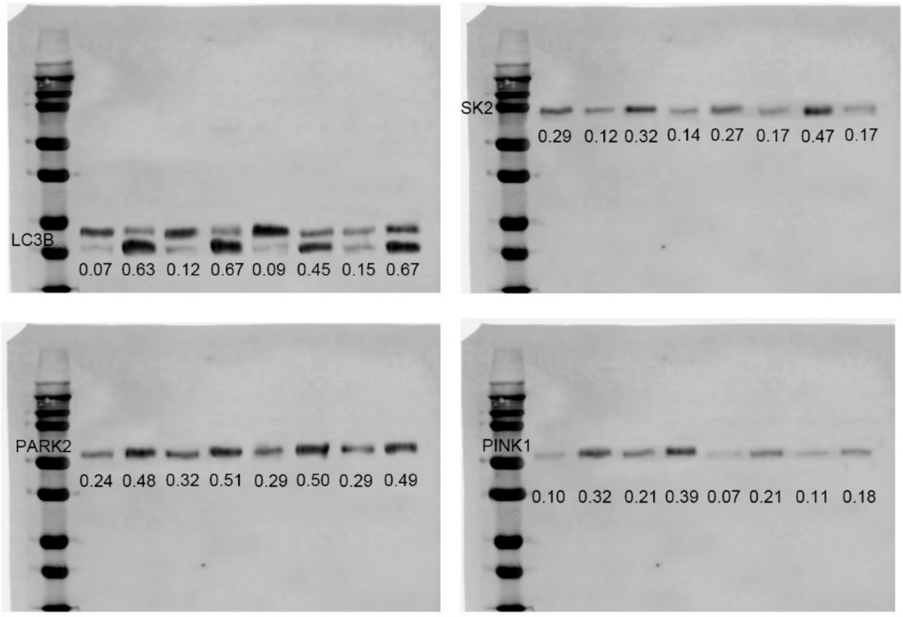

Figure S6. The raw data and the intensity ratio of Figure 2E.

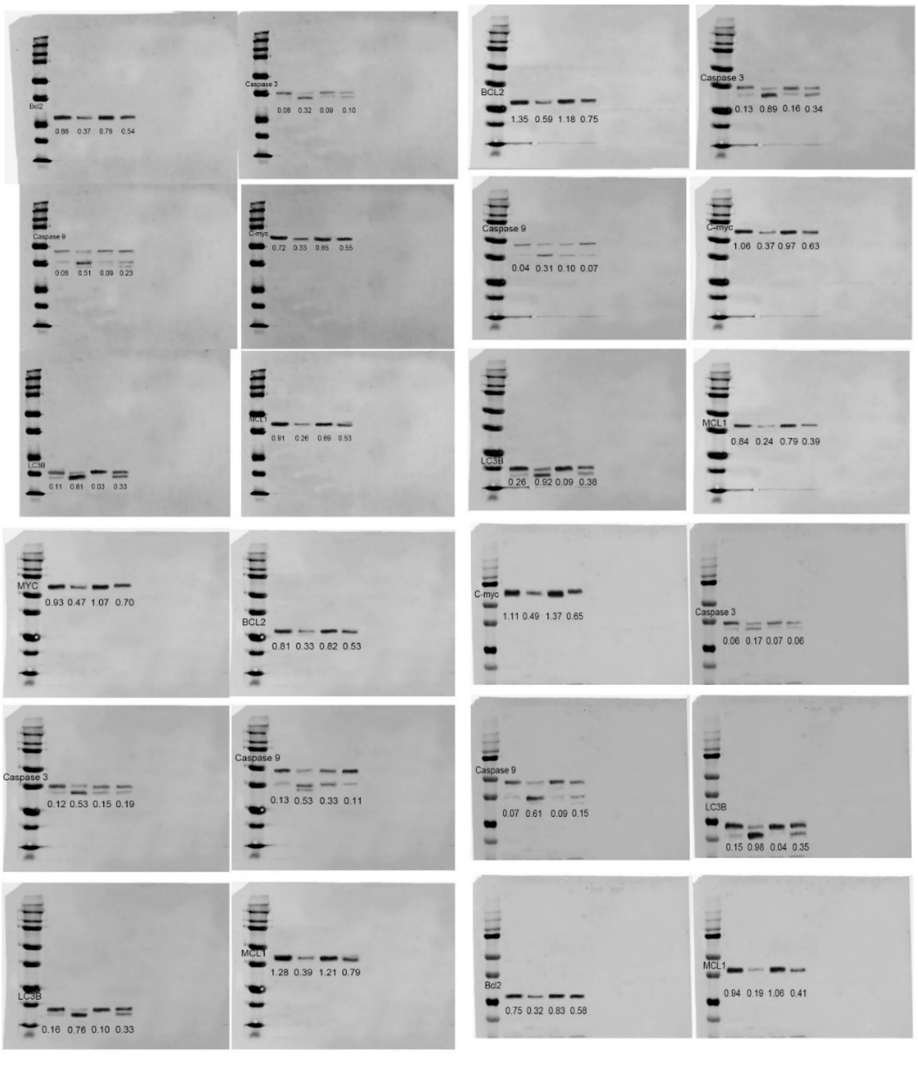

Figure S7. The raw data and the intensity ratio of Figure 3.

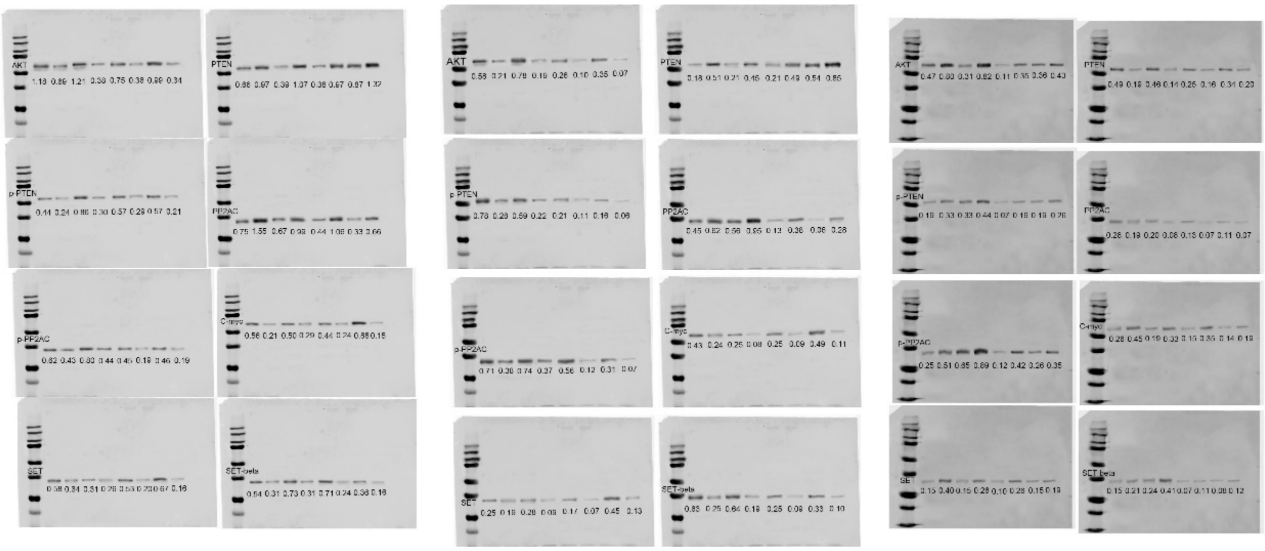

Figure S8. The raw data and the intensity ratio of Figure 4 and Figure S4.

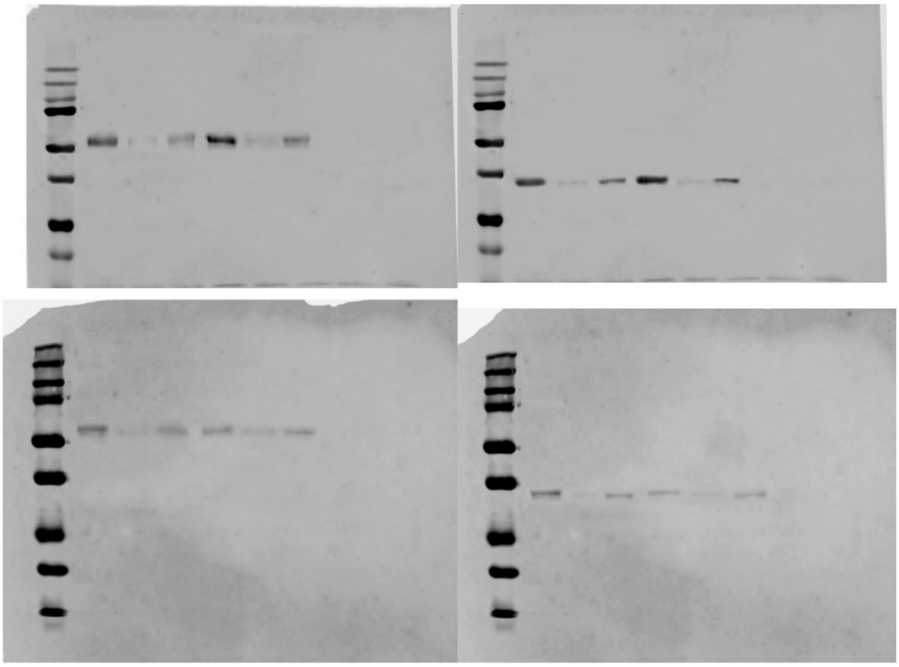

Figure S9. The co-IP raw data of Figure 4D.

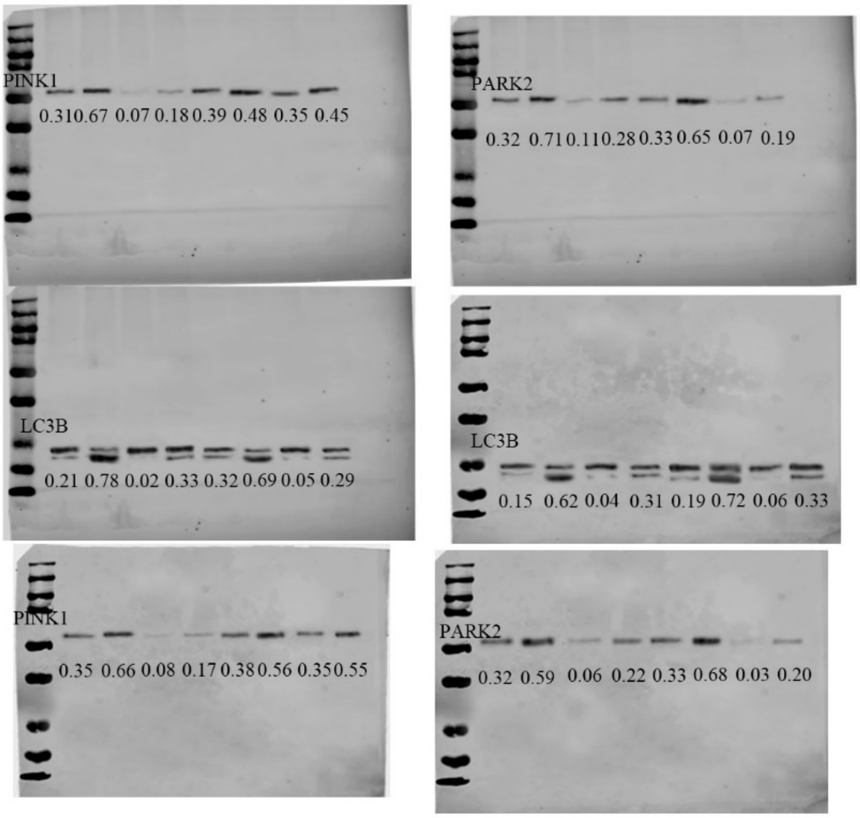

Figure S10. The raw data and the intensity ratio of Figure 6.
